# Confronting Ghana's Hypertension Crisis: Epidemiology, Barriers, and Policy Pathways

**DOI:** 10.1002/puh2.70410

**Published:** 2026-10-05

**Authors:** Michael Asiedu Owiredu, Obaa Yaa Asantewaa Korsah, Margaret Addo, Maame Afua Korsah, Sule Apaame, Seth Offei Addo

**Affiliations:** ^1^ Seventh Day Adventist Valley View Hospital Oyibi Ghana; ^2^ New Edubiase Government Hospital New Edubiase Ghana; ^3^ Gbawe Seventh‐Day Adventist Hospital Gbawe Ghana; ^4^ Kaneshie Polyclinic Accra Ghana; ^5^ Department of Parasitology Noguchi Memorial Institute for Medical Research University of Ghana Accra Ghana

**Keywords:** Ghana, health systems strengthening, hypertension, non‐communicable diseases, primary health care

## Abstract

One of Ghana's most significant yet under‐recognized public health issues is hypertension, which is driving, increases in cardiovascular disease, disability and premature mortality. Despite ongoing improvements in primary healthcare and increased access through the National Health Insurance Scheme, the prevalence of hypertension remains high, and knowledge, treatment and management levels remain unacceptably low. In this perspective, we examine Ghana's hypertension crisis from three interrelated perspectives: the changing epidemiology and population distribution of hypertension; the barriers to effective prevention and long‐term control at the individual, community and health‐system levels; and priority policy and implementation pathways for improving hypertension management. Drawing on data from regional studies, national surveys and facility‐based research, we argue that Ghana's hypertension burden reflects broader structural changes linked to urbanization, dietary change and the ongoing fragmentation of the health system. We argue that present trends cannot be reversed by relying simply on fragmented, episodic hypertension care models that prioritize treatment over holistic prevention, follow‐up and long‐term management. To achieve long‐term progress, primary health care must be strengthened through the implementation of the World Health Organization's (WHO) HEARTS Technical Package, which includes standardized treatment protocols, reliable access to essential medicines and validated blood pressure devices, team‐based care, population‐level prevention strategies and robust health information systems. Effectively managing hypertension is consequently crucial for lowering cardiovascular morbidity and mortality and for demonstrating Ghana's ability to respond fairly and sustainably to the expanding problem of non‐communicable diseases.

## 1 Introduction

Cardiovascular disorders are the largest cause of non‐communicable diseases (NCDs), which currently account for the majority of fatalities worldwide [1, 2]. Ghana and other low‐ and middle‐income countries (LMICs) are going through a fast epidemiological transition marked by an increase in chronic illnesses and a decrease in the mortality from infectious diseases [3]. For health systems traditionally built to treat acute illnesses rather than long‐term care, this change presents significant challenges. At the core of this shift is hypertension. Hypertension, the most significant modifiable risk factor for cardiovascular disease, is a major contributor to the global burden of stroke, ischaemic heart disease, heart failure and chronic kidney disease [4, 5]. However, until problems develop, it is essentially asymptomatic, gaining the alias ‘silent killer’ [6]. Despite being comparatively underprioritized in public health planning, financing and surveillance, hypertension has quietly emerged as one of the most prevalent chronic illnesses among adults [7]. In Ghana, approximately 6% of all persons with hypertension have regulated blood pressure, 22% are on medication, and 35% are aware of their condition [8, 9]. Hypertension is the primary risk factor for stroke, which is one of the major causes of adult mortality and long‐term disability [10]. These patterns put rising financial strain on households and the healthcare system, and they pose a threat to life expectancy. Ghana's response to hypertension is still disjointed in spite of these findings. Infectious diseases, maternal and child health, and episodic health risks have traditionally been given priority in health policies and investments, but NCDs have received very little attention. Despite Ghana's adoption of national NCD plans, their execution has been inconsistent and underfunded [11]. As a result, hypertension is usually recognized late, managed inefficiently and poorly controlled.

The World Health Organization's (WHO) HEARTS Technical Package has become recognized as the international standard for improving hypertension prevention and control in primary care. HEARTS advocates for five interconnected components: standardized treatment protocols, consistent access to essential medicines and validated blood pressure devices, team‐based care through task sharing, patient‐centred services that are accessible and affordable, and information systems that support monitoring and quality improvement [12, 13]. Assessing Ghana's hypertension response using this approach provides an evidence‐based road map for improving hypertension care at scale. This perspective suggests that rather than being solely the result of individual behaviour, Ghana's hypertension crisis is a reflection of deeper societal, environmental and health system factors. To address it, fragmented, episodic hypertension care must evolve into unified, longitudinal primary health care supported by established treatment regimens, routine blood pressure measurement, dependable medication supply and ongoing patient follow‐up.

## 2 The Hidden Crisis: Prevalence and Patterns of Hypertension in Ghana

In Ghana, hypertension has emerged as one of the most common chronic illnesses. According to national estimates, 25%–35% of persons over the age of 18 have hypertension, and the prevalence rises sharply with age [8]. Prevalence often exceeds 50% among persons aged 50 and older, underscoring the mounting burden of population ageing [7]. Hypertension patterns have been profoundly influenced by urbanization. Urban populations generally display higher prevalence than rural residents, suggesting lifestyle changes such as lower physical activity, increasing intake of processed foods and rising obesity rates [14]. However, as rural populations increasingly embrace sedentary lifestyles and meals high in energy, recent research indicates that the gap between rural and urban areas is closing [15]. This convergence highlights how widespread Ghana's hypertension crisis is. Social and behavioural dynamics are further demonstrated by sex differences. Men have lower awareness and worse health‐seeking behaviour, whereas women frequently show higher crude prevalence, in part because of higher obesity prevalence [8]. These trends delay diagnosis and increase the risk of men developing complications, including heart failure and stroke. The most significant issue with hypertension in Ghana is not just how prevalent it is, but also how challenging the healthcare system is for patients to navigate across the continuum of hypertension diagnosis, treatment and long‐term management. A significant portion of the population with hypertension remains unaware of their condition [8, 16]. Effective blood pressure control is rare (typically less than 20%), and treatment coverage is uneven among those who are diagnosed [16, 17]. These gaps show shortcomings in long‐term adherence, continuity of care and opportunistic screening. The consequences are increasingly visible. Stroke has emerged as a leading cause of adult mortality and disability in Ghana, placing substantial pressure on tertiary health facilities and families [18]. Admissions for hypertensive heart disease and chronic kidney disease are rising, with significant economic implications. Hypertension also clusters with other cardiometabolic risk factors, including obesity, diabetes and harmful alcohol use, amplifying cardiovascular risk [19].

## 3 Intersecting Barriers to Hypertension Prevention and Control

Intersecting barriers that operate at the individual, community, health system and policy levels impede the prevention and treatment of hypertension in Ghana. Misconceptions and a lack of awareness about hypertension are still common at the individual level. Both awareness of hypertension and willingness to seek adequate biomedical care are significantly impacted by level of education [20]. As a result, some people with hypertension use herbal medicines, frequently in conjunction with prescription drugs, which reflects deeply ingrained cultural beliefs and a lack of faith in hospital‐based therapies [21]. Medication prices, perceived or actual side effects and inconsistent drug availability are other factors that limit the adherence to antihypertensive therapy [22, 23]. Interestingly, even patients covered by the National Health Insurance Scheme (NHIS) regularly experience shortages of necessary antihypertensive medications in public institutions, which can lead to out‐of‐pocket costs or treatment interruptions [24]. Community‐level variables that influence cardiovascular risk exacerbate these individual‐level difficulties. Rapid urbanization has changed social and physical surroundings, reducing opportunities for physical activity due to poorly planned metropolitan areas and sedentary job habits. Simultaneously, there has been a rise in the prevalence of dietary shifts towards highly processed, energy‐dense and salt‐rich foods, which increases the risk of hypertension [14]. Effective management of hypertension is nevertheless hampered by persisting systemic flaws in the health system. In addition to providing services that are frequently financially and physically inaccessible, especially for rural and peri‐urban populations, public health facilities struggle to maintain a consistent supply of antihypertensive drugs and deal with a dearth of qualified healthcare workers [25]. These limitations weaken the hypertension care cascade and compromise continuity of care.

Limitations at the policy level exacerbate these difficulties. In Ghana, NCD programmes have traditionally received insufficient domestic funding and little political attention. Despite the development of national NCD policies, their execution is still inadequate, heavily depending on pilot projects sponsored by donors and lacking accountability systems to guarantee sustainability and scale‐up [26]. Inadequate hypertension prevention, treatment and control are sustained by a reinforcing loop created by these complex and interconnected impediments.

## 4 Policy and Implementation Pathways for Action

To address Ghana's growing hypertension burden, primary healthcare must implement coordinated strategies that improve prevention, treatment and long‐term management. The WHO HEARTS technical package offers a practical framework for improving hypertension control using evidence‐based therapies [13]. Population‐level treatments should incorporate evidence‐based salt reduction measures. Potassium‐enriched salt alternatives have been shown to improve cardiovascular health, but they must be carefully evaluated in Ghana due to cost, dietary patterns, and the incidence of chronic renal disease [27, 28]. Front‐of‐pack warning labels have lowered sodium purchases and prompted food reformulation in Latin America, which supports their use in Ghana [29]. Public education initiatives promoting healthy diets, regular exercise, quitting smoking and limiting alcohol intake are all equally crucial [12, 13]. Standardized, evidence‐based treatment strategies should be used at all levels of care to improve diagnosis, treatment start and blood pressure control. The NHIS must be strengthened to enable affordable access to important hypertension medications, approved blood pressure devices and routine follow‐up in order to improve treatment adherence and reduce financial barriers [30]. Team‐based care and duty shifting might increase access by allowing trained community health nurses and other non‐physician healthcare workers to conduct screenings, commence treatment using standardized protocols and offer follow‐up care, especially in impoverished areas. Primary care should remain the foundation of hypertension management, supplemented by universal blood pressure measurement for every adult attending a health facility and integrated chronic illness services [31]. Finally, advanced health information systems and patient registries are required to track treatment outcomes, improve continuity of care and guide evidence‐based policymaking. Implementing these interconnected initiatives within the WHO HEARTS framework will significantly enhance hypertension control, prevent cardiovascular disease and speed up progress towards universal health coverage and the Sustainable Development Goals [13].

## 5 Conclusion

Hypertension is a major public health concern in Ghana. Its high prevalence, low awareness and poor control indicate systemic gaps rather than a dearth of effective solutions. Integrated, equity‐focused approaches that support basic healthcare, empower communities and match policy to the realities of managing chronic illnesses are needed to address this challenge. Ghana may significantly lower the burden of cardiovascular disease and improve its ability to address the growing problem of NCDs by making hypertension a priority in national development and universal health coverage agendas.

## Author Contributions

**Maame Afua Korsah**: investigation, writing – review and editing. **Michael Asiedu Owiredu**: investigation, writing – review and editing. **Sule Apaame**: investigation, writing – review and editing. **Margaret Addo**: investigation, writing – review and editing. **Obaa Yaa Asantewaa Korsah**: investigation, writing – review and editing. **Seth Offei Addo**: conceptualization, investigation, writing – original draft.

## Funding

The authors have nothing to report.

## Ethics Statement

The authors have nothing to report.

## Conflicts of Interest

The authors declare no conflicts of interest.

## Data Availability

No new data were generated for this article.
